# Involvement of NF-κBIZ and related cytokines in age-associated renal fibrosis

**DOI:** 10.18632/oncotarget.14614

**Published:** 2017-01-12

**Authors:** Ki Wung Chung, Hyeong Oh Jeong, Bonggi Lee, Daeui Park, Dae Hyun Kim, Yeun Ja Choi, Eun Kyeong Lee, Kyung Mok Kim, June Whoun Park, Byung Pal Yu, Hae Young Chung

**Affiliations:** ^1^ Department of Pharmacy, College of Pharmacy, Pusan National University, Busan, 46241, Republic of Korea; ^2^ Systems Toxicology Research Center, Korea Institute of Toxicology, Daejeon, 34114, Republic of Korea; ^3^ Department of Physiology, The University of Texas Health Science Center at San Antonio, San Antonio, TX, 78229, USA

**Keywords:** NF-?BIZ, aging, inflammation, renal fibrosis

## Abstract

Chronic inflammation is a major contributor to age-related nephropathic changes, including renal fibrosis. In this study, various experimental paradigms were designed to delineate the role played by NF-?BIZ (also known as I?B?) in age-associated renal fibrosis. Analyses based on RNA-sequencing findings obtained by next generation sequencing (NGS) revealed the upregulations of NF-?BIZ and of IL-6 and MCP-1 (both known to be regulated by NF-?BIZ) during aging. The up-regulation of NF-?BIZ in aged rat kidneys coincided with increased macrophage infiltration. In LPS-treated macrophages, oxidative stress was found to play a pivotal role in NF-?BIZ expression, suggesting age-related oxidative stress is associated with NF-?BIZ activation. Furthermore, these *in vitro* findings were confirmed in LPS-treated old rats, which showed higher levels of oxidative stress and NF-?BIZ in kidneys than LPS-treated young rats. Additional *in vitro* experiments using macrophages and kidney fibroblasts demonstrated NF-?BIZ and related cytokines participate in fibrosis. In particular, increased levels of NF-?BIZ-associated cytokines in macrophages significantly up-regulated TGF-β induced kidney fibroblast activation. Moreover, experiments with NF-?BIZ knocked down macrophages showed reduced TGF-β- induced kidney fibroblast activation. The findings of the present study provide evidence regarding an involvement of NF-?BIZ in age-associated progressive renal fibrosis and provides potential targets for its prevention.

## INTRODUCTION

Chronic inflammation is a major risk factor that underlies various chronic diseases [1]. Although the inflammatory process is a normal, acute, defensive response to infection or tissue damage, unresolved low-grade chronic inflammation exacerbates age-related diseases and the effects of aging at the molecular level *via* the NF-kB (nuclear factor kappa-light-chain-enhancer of activated B cells) pathway, as delineated by the molecular inflammation hypothesis [2]. This hypothesis provides insight of the oxidative stress-induced molecular pathway and links age-related physiological changes and the pathogenesis of many age-related diseases. Age-related inflammation has several characteristic features, such as, dysregulation of the immune systems and increased oxidative stress, and dysregulation of the immune system is primary cause of inflammation during aging [3, 4]. Dysregulation of the innate immune system is characterized by persistent inflammatory responses involving multiple immune and non-immune cells. Redox imbalance during aging also insidiously increases inflammation due to the continuous production of reactive oxygen species (ROS). Evidence strongly suggests that chronic up-regulation of NF-κB induced by immune system dysregulation and aberrant oxidative stresses provokes the expression of pro-inflammatory mediators, like TNF-α, IL-1β, IL-6, COX-2, and iNOS, during the aging process.

NF-κB inhibitor zeta (NF-κBIZ, also known as MAIL and IκBζ), which is encoded by *NFKBIZ*, is an atypical nuclear member of the IκB family [5]. NF-κBIZ has been recently implicated in differential NF-κB target gene expression in immune cells, although its physiological function remain largely unknown [6]. Unlike other classical IκB proteins that are constitutively expressed and controlled by degradation, NF-κBIZ expression is barely detectable in unstimulated cells, but when cells are stimulated with various inflammatory stimuli, it is rapidly induced [5]. NF-κBIZ has been suggested to regulate NF-κB signaling, as reporter analyses indicated NF-κBIZ may act as an inhibitor of NF-κB [7]. In contrast, other studies have demonstrated that NF-κBIZ can induce the gene expressions of individual genes targeted by NF-κB, such as, IL-6 and MCP-1 in macrophages [8, 9]. However, its role in aged nephropathic conditions has not been explored to date.

Progressive renal fibrosis is the final pathway common to all renal diseases that ultimately lead to end-stage renal failure [10, 11], a devastating condition requiring dialysis or kidney transplantation [12]. The presence of progressive renal fibrosis manifests as reduced excretory function (reduced glomerular filtration rate) and elevated urinary protein excretion [13]. It has been well established that renal aging is associated with structural changes in kidneys, such as, glomerulosclerosis, interstitial fibrosis, and tubular atrophy [14, 15]. Aging disturbs normal renal structure-function relationships for many reasons, which include the dysregulation of cellular energy sensors, oxidative stress, mitochondrial dysfunction, and abnormal immune functions that compromise nephron structural integrity with progressive renal fibrosis [16]. Recently, renal aging was highlighted in an investigation of renal structural changes in healthy kidney donors [17]. The authors observed subclinical age-related nephropathy on implantation renal biopsy of donors, which was undetectable by conventional clinical diagnostic testing. The investigators concluded that age-related structural changes in the kidney occur earlier than they previously expected [17]. Of the many fibrosis-driving signals, inflammation is a common feature of many fibrosis-related diseases [18].

Although the roles of NF-κBIZ have been investigated in many immune cells, its physiological roles have not been as extensively addressed. In the present study, we sought to delineate the role played by NF-κBIZ in age-associated renal fibrosis using systems biological and biochemical approaches. In addition, because aberrant oxidative stresses are known to be associated with the aging process, we investigated the role of oxidative stress on NF-κBIZ activation. Based on our systems biology results and biologic experimental results, we examined the role played by NF-κBIZ in age-related progressive renal fibrosis.

## RESULTS

### The importance of the NF-κBIZ gene by RNA sequencing analysis

The statistical significance of genes related to the NF-κB family was tested by RNA-Seq analysis of NGS data as determined in previous studies [19] and Materials and Methods (Supplementary Table 1). Of the NF-κB family members, including IκB family and IκB kinase (IKK) complex, NF-κBIZ was the only gene found to be significantly up-regulated in aged rats kidneys (3.305 fold up-regulated; Q value = 0.00577; Supplementary Table 1).

Because NF-κBIZ was recently implicated in differential NF-κB target gene expression in immune cells, we analyzed 360 target genes of NF-κB in the Dr. Thomas Gilmore database (http://www.bu.edu/nf-kb/gene-resources/target-genes). We found 54 of these genes were differentially expressed in young and old male Sprague Dawley (SD) rat kidneys (Q value < 0.05, Supplementary Table 2). Furthermore, of these 54 genes, 51 were up-regulated in aged kidneys, suggesting the importance of NF-κB and of inflammation in the aging process (Supplementary Table 3, Supplementary Figure 1). Surprisingly, IL-6 and MCP-1, which are known to be regulated by NF-κBIZ, were also significantly up-regulated in old rats as compared with young rats (6.761 fold up-regulated, Q value = 0.008, 3.599 fold up-regulated, Q value = 0.001 respectively, Supplementary Table 3). In addition, IL-6 receptor was also significantly up-regulated by aging by 2.908 fold (Q value = 0.013, Supplementary Table 3). These observations indicate NF-κBIZ and its related genes were significantly up-regulated.

### Validation of RNA-sequencing data by biochemical analysis

**Figure 1:**
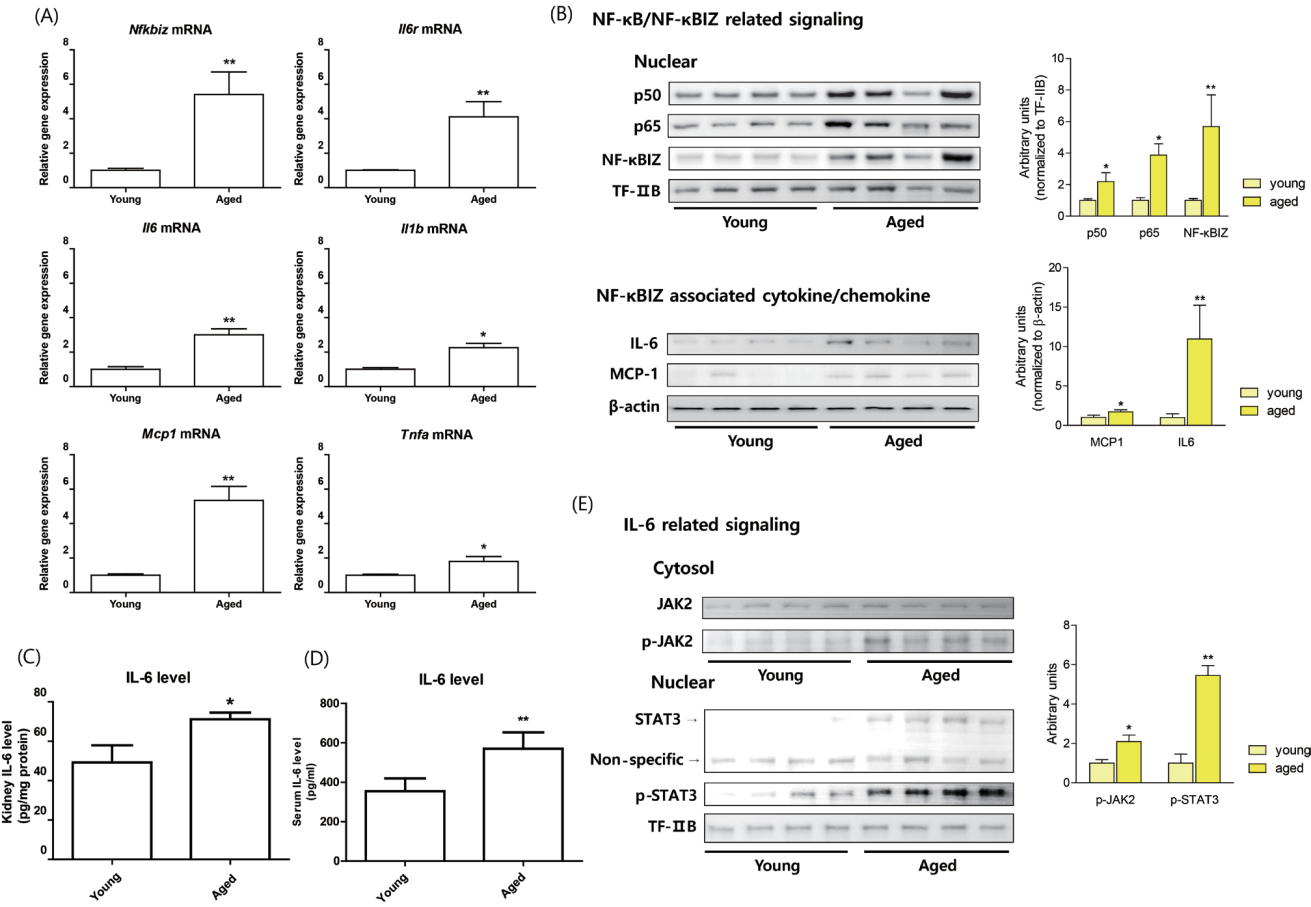
Increased NF-κBIZ and NF-κBIZ-related signaling in aged rat kidney Young (6 month) and old (24 month) rat kidneys were used to validate NGS data. **A**. The mRNA expressions of *Nfkbiz*, *Il6*, *Mcp1*, *Il6r*, *Tnfa*, and *Il1b* were measured by qPCR in young and old rat kidneys (*n* = 6). Results were normalized *vs*. *Gapdh*. Data are expressed as means ± SEMs. *P < 0.05 *vs*. the young group. **P < 0.01 *vs*. the young group. **B**. The protein levels of NF-κB family members and of NF-κBIZ related proteins were detected in young and old rat kidneys. Western blots were performed to estimate the nuclear protein levels of p50, p65, and NF-κBIZ in nuclear fractions of old kidneys. TF-IIB was used as a loading control. NF-κBIZ related cytokines (IL-6, MCP-1) were detected by Western blotting. β-Actin was used as the loading control. **C**. Kidney IL-6 levels were determined by ELISA. Data are expressed as means ± SEMs. **P < 0.01 *vs*. the young group. **D**. Serum IL-6 levels were determined by ELISA. Data are expressed as means ± SEMs. **P < 0.01 *vs*. the young group. **E**. IL-6 downstream signaling pathway (JAK-STAT pathway) were detected in young and old rat kidneys. Western blotting was used to assess the protein levels of JAK2 and phosphorylated-JAK2 in cytosolic fractions of aged kidneys, and Stat3 and phosphorylated-Stat3 in nuclear fractions of aged kidneys. TF-IIB was used as the loading control.

To confirm age-related NF-κB target genes expression using RNA-Seq data, quantitative PCR was performed. The genes were selected based on associations with NF-κBIZ or with other well-known target genes of NF-κB. As shown in Figure 1, NF-κBIZ and its related genes (IL-6, MCP-1, and IL-6R) were all significantly up-regulated during renal aging. TNFα and IL-1β were also up-regulated, but to a lesser extent than NF-κBIZ related genes (Figure 1A). These results indicate the importance of NF-κBIZ and its related proteins, and confirm RNA-Seq data from NGS. To further confirm the role of increased NF-κBIZ in aged kidneys, the protein expression of NF-κBIZ and its related cytokine changes were examined in young and old rat kidneys. In agreement with previous results [20], nuclear p65 and p50 protein levels increased with aging (Figure 1B). In addition, the expression of NF-κBIZ protein was elevated in the nuclear fractions of aged kidneys, which concurred with qPCR data (Figure 1B). Furthermore, the protein levels of NF-κBIZ related cytokines were also up-regulated in aged kidney (Figure 1B, 1C). Serum IL-6 levels were also increased in aged rat (Figure 1D). To further assess the effects of increased levels of NF-κBIZ-related cytokines, the activation of IL-6 signaling was investigated in young and old rat kidneys. The activation of IL-6 by its receptor leads to the phosphorylation of JAK2 and the nuclear translocation of STAT3. Aged kidneys showed increased JAK2 phosphorylation and significantly higher levels of STAT3 and of phosphorylated STAT3 in nuclear fractions (Figure 1E). These findings confirm that the up-regulations of NF-κBIZ and of its related cytokines induces IL-6, and thus, upregulates the JAK2/STAT3 signaling pathway in the kidneys of aged rats.

### NF-κBIZ in aged kidneys and macrophage infiltration

**Figure 2:**
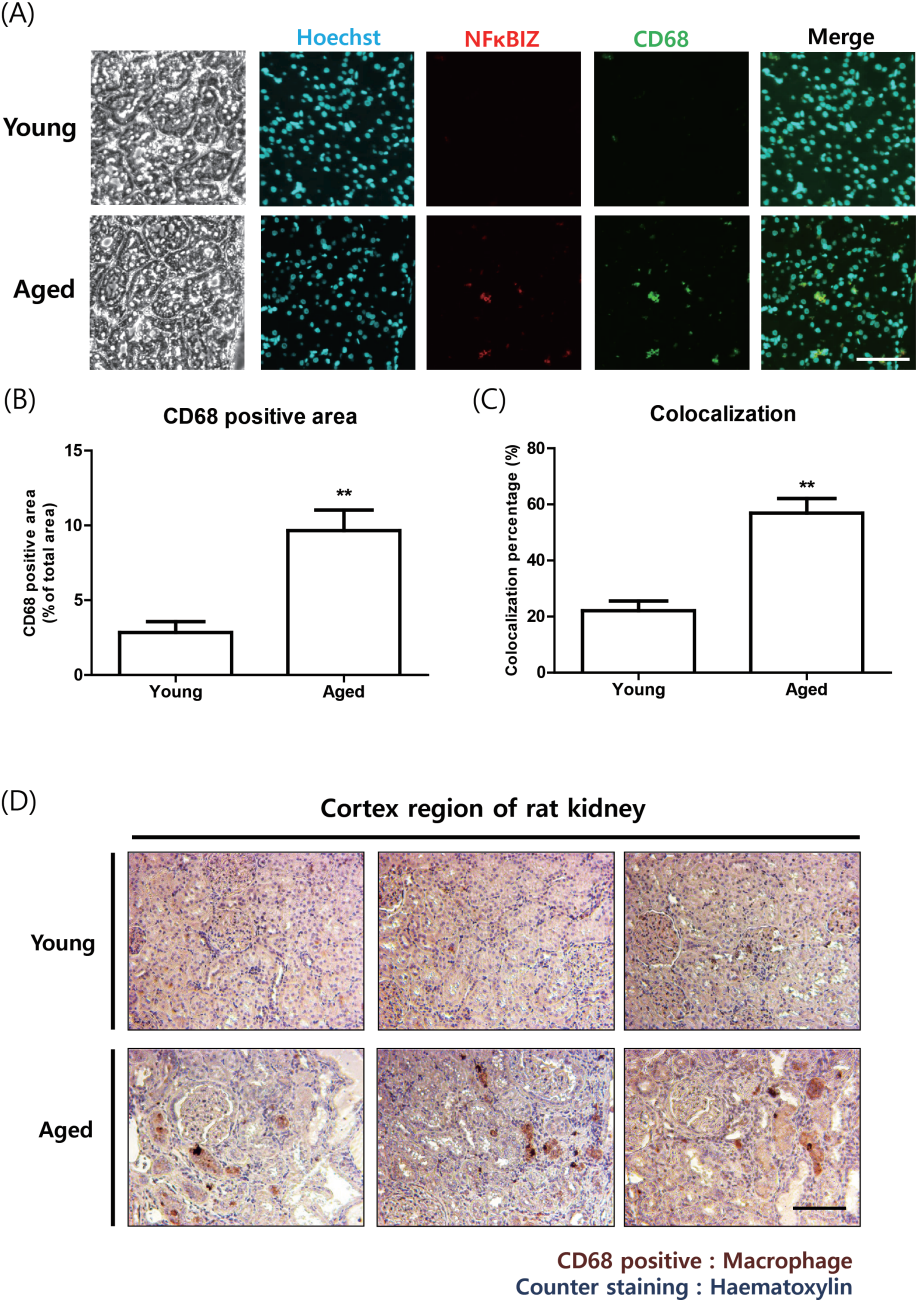
Increased NF-κBIZ protein levels were associated with macrophage infiltration in aged rat kidneys **A**. NF-κBIZ and macrophage marker protein CD-68 were detected using two primary antibodies. NF-κBIZ antibody and CD-68 antibody were simultaneously administered to young and old kidney paraffin sections for double immunofluorescence staining. Tissue sections were counterstained with Hoechst33342. After staining, fluorescence was observed by confocal microscopy. Scale bar = 200 μm. **B**. CD68 positive area from immunofluorescence staining were quantified. **P < 0.01 *vs*. the young group. **C**. Colocalization percentage was calculated based on immunofluorescence staining data. **P < 0.01 *vs*. the young group. **D**. Increased macrophage infiltration in old kidneys was confirmed by immunostaining kidney cortex sections for CD-68 (brown); slides were counterstained with haematoxylin (blue). The upper pictures represent young kidneys and the lower pictures aged kidneys. Scale bar = 100 μm.

To examine whether increased NF-κBIZ is associated with the infiltration of immune cells during aging, we performed immunohistological analysis. Macrophage marker protein CD-68 and NF-κBIZ were found to colocalize in aged rat kidneys by immunofluorescence (Figure 2A). CD-68 positive area as well as colocalization between CD-68 and NF-κBIZ was increased in aged rat kidneys (Figure 2B, 2C). Immunohistochemistry showed increased macrophage infiltration in old rats (Figure 2D). These data suggest that increased NF-κBIZ during aging is associated with increased infiltration of macrophages during aging.

### Modulation of IL-6/MCP-1 by NF-κBIZ in LPS-treated macrophages

*In vitro* experiments were conducted to further assess the role of NF-κBIZ. Because NF-κBIZ were elevated in the macrophages of old rats, peritoneal derived macrophages were utilized to confirm the previously discovered role. LPS (500 ng/ml) treatment rapidly induced an increase in the protein level of NF-κBIZ peritoneal macrophages (Supplementary Figure 2A), and increased *Nfkbiz* mRNA expression (Supplementary Figure S2B). Next, we examined the mRNA levels of several cytokines known to be transcribed by NF-κB activation. LPS treatment induced the up-regulations of *Il1b* and *Tnfa* mRNA rapidly, and of *Mcp1* and *Il6* rather later (Supplementary Figure 2B). These observations concur with previous reports that NF-κBIZ-related genes are induced in a secondary manner [8, 9].

To confirm the role played by NF-κBIZ in the inductions of specific cytokines, a NF-κBIZ knock-down experiment was conducted using siRNA. *Nfkbiz* siRNA (2.5 nM) significantly reduced the mRNA and protein expressions in peritoneal macrophages (Supplementary Figure S3A, S3B). In addition, when NF-κBIZ was knocked-down, the mRNA expression levels of *Il6* and *Mcp1* induced by LPS were significantly reduced (Supplementary Figure 3C). However, the mRNAs of *Il1b* and *Tnfa* were not influenced by NF-κBIZ siRNA, indicating these cytokines are not associated with NF-κBIZ (Supplementary Figure 3C). Collectively, these results confirm the previously described role of NF-κBIZ and demonstrate NF-κBIZ participates in the inductions of IL-6 and MCP-1.

### Effects of oxidative stress on the induction of NF-κBIZ in macrophages by LPS

**Figure 3:**
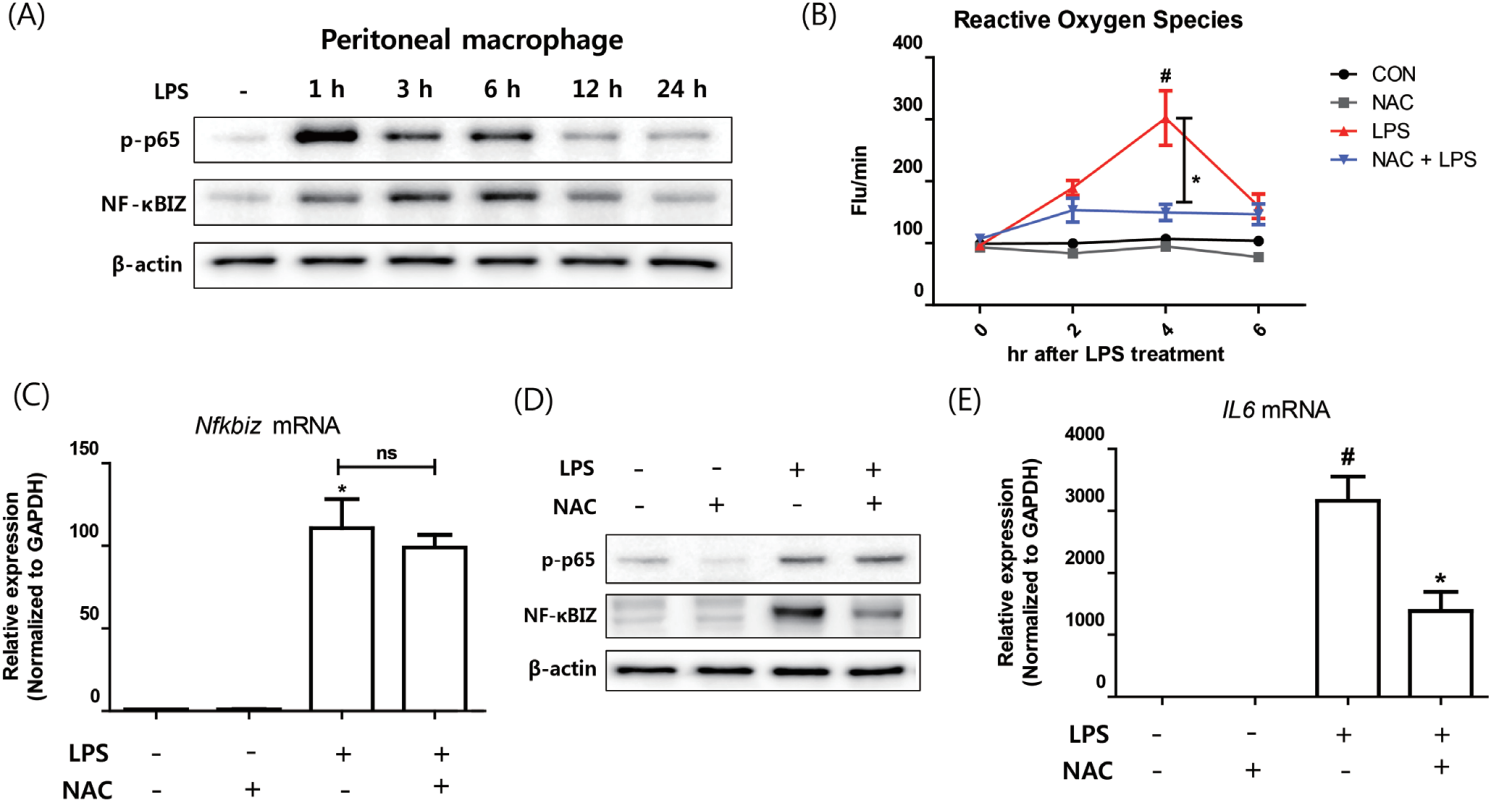
Effect of oxidative stress on NF-κBIZ activation in macrophages **A**. Western blotting was performed to examine the time-dependent expression of NF-κBIZ and p65 activation in LPS treated macrophages. β-Actin was used as the loading control. **B**. Oxidative stresses were measured using DCFDA in LPS-treated macrophages pretreated or not with NAC. Data are expressed as means ± SEMs. *P < 0.05 *vs*. non-treated macrophages. #*P* < 0.05 *vs*. LPS treated macrophages. **C**. *Nfkbiz* mRNA levels were assessed by qPCR in LPS-treated macrophages pretreated with or without NAC. Results were normalized *versus* *Gapdh*. *P < 0.05 *vs*. non-treated macrophages. **D**. Western blotting was performed to investigate the effects of NAC on the protein expression of NF-κBIZ and activation of p65 in LPS treated macrophages. β-Actin was used as the loading control. **E**. mRNA expressions of *Il6* were measured by qPCR in LPS-treated macrophages pretreated with or without NAC. Data are expressed as means ± SEMs. *P < 0.05 *vs*. non-treated macrophages. #*P* < 0.05 *vs*. LPS treated macrophages.

As oxidative stress is causally linked with inflammation and aging, and plays an important role as a second signaling messenger [2, 21], we examined the role of oxidative stress on NF-κBIZ activation further. Treatment of macrophages with LPS induced p65 activation (p65 phosphorylation) and increase NF-κBIZ protein levels (Figure 3A), which were sustained through 12 h after LPS treatment (Figure 3A). LPS also increased oxidative stress as determined by ROS levels, which peaked after 4 h of LPS treatment (Figure 3B). To determine whether the scavenging of oxidative stress suppresses NF-κBIZ expression, we pretreated macrophages with N-acetyl cysteine (NAC; a well-known ROS scavenger), and found NAC pretreatment significantly reduced LPS-induced oxidative stress (Figure 3B). Interestingly, NAC did not affect the activation of p65 (as determined by p65 phosphorylation) or up-regulation of NF-κBIZ mRNA by LPS (Figure 3C, 3D). However, NAC pretreatment decreased NF-κBIZ protein levels in macrophages (Figure 3D), and reduced the IL-6 mRNA expression (Figure 3E). These results indicate that oxidative stress is associated with the LPS-induced up-regulation of NF-κBIZ protein in macrophages.

### LPS-induced NF-κBIZ activation during aging

**Figure 4:**
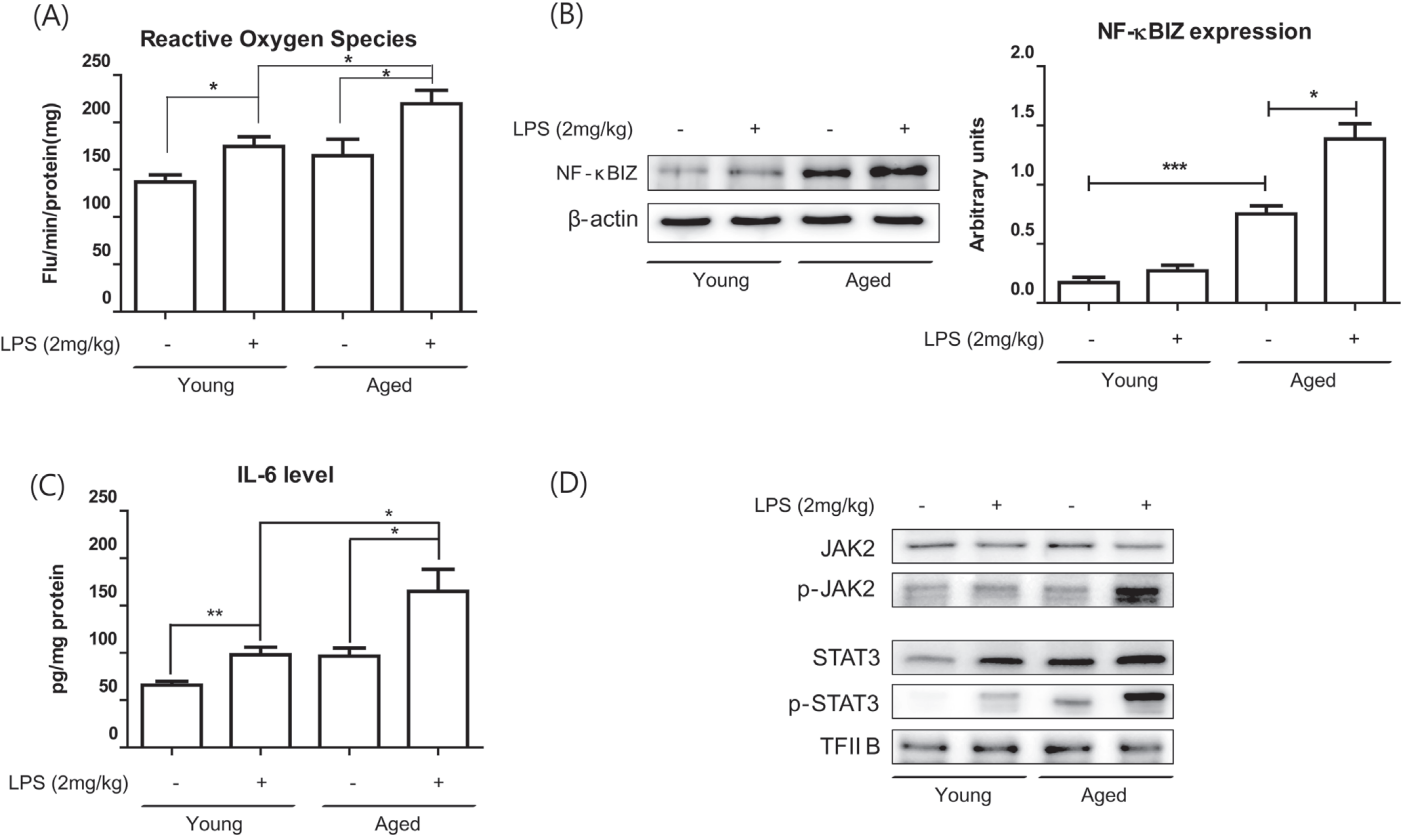
Aging potentiated LPS-induced NF-κBIZ activation LPS (2 mg/kg) was administered to young and old rats to determine the effects of aging on LPS-induced oxidative stress increased and NF-κBIZ activation. **A**. Oxidative stress was measured using DCFDA in young and old rat kidney homogenates pretreated with or without LPS. Data are expressed as means ± SEMs. *P < 0.05. **B**. Western blotting was performed to assess NF-κBIZ protein levels in the kidneys of LPS-treated young and old rats. β-Actin was used as the loading control. **C**. IL-6 levels in young and old rat kidney homogenates were determined by ELISA. Data are expressed as means ± SEMs. *P < 0.05. **P < 0.01. **D**. IL-6 downstream signaling pathway (JAK-STAT pathway) were detected in the kidneys of young and old rats treated with or without LPS. Western blotting was performed to assess JAK2 and phosphorylated-JAK2 protein levels in cytosolic fractions of aged kidneys, and Stat3 and phosphorylated-Stat3 in nuclear fractions of aged kidneys. TF-IIB was used as the loading control.

To investigate the effects of oxidative stress and NF-κBIZ during aging, we checked the effects of aging on inflammation-induced oxidative stress and NF-κBIZ activation in kidney tissues. Young and old rats were administered LPS (2 mg/kg), which has been extensively used in studies on inflammation. Aged rat kidneys showed significantly higher basal levels of oxidative stress (Figure 4A), and higher increases in oxidative stress by LPS treatment (Figure 4A). In addition, NF-κBIZ and IL-6 protein levels were also higher in aged rat kidneys (Figure 4B, 4C) when determined by Western blot and ELISA respectively. These increases were followed by an increase in the downstream signaling of IL-6 (Figure 4D). STAT3 phosphorylation was highly induced only in aged rat against LPS (Figure 4D). These findings suggest the possible role of oxidative stress and NF-κBIZ in the response differences against LPS in young and aged rat kidney.

### The relation between age-associated renal fibrosis and increased NF-κBIZ-related signaling

**Figure 5:**
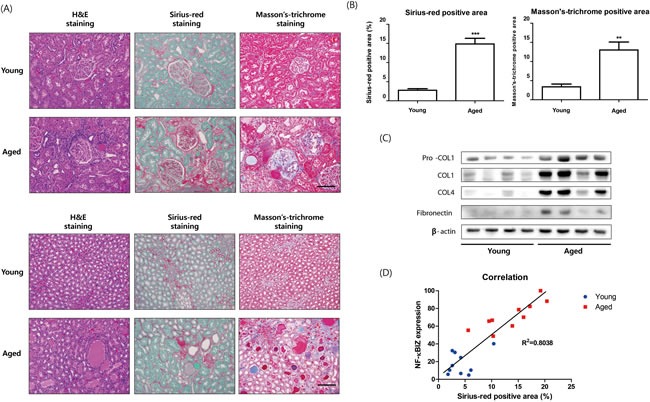
Relation between age-associated renal fibrosis and increased NF-κBIZ related signaling **A**. Aging increased fibrosis in kidney cortices and medullae. Young and aged kidney cortices and medullae were histologically stained for fibrosis. H&E staining, Sirius-red staining, and Masson's trichrome staining showed more renal fibrosis in the cortices of aged rat kidneys. Fibrosis scores were assigned based on Sirius-red staining intensities in kidney cortices (*n* = 6). Scale bar = 100 μm. **B**. Fibrotic areas were calculated based on Sirius-red and Masson's Trichrome staining. **C**. Aging increased the levels of fibrosis-related proteins. Western blotting was performed to assess the nuclear protein levels of pro-collagen1, collagen1, pro-collagen3, and fibronectin. β-Actin was used as loading control. **D**. Correlation between NF-κBIZ activation and fibrosis extent. The graph shows the correlation observed between NF-κBIZ (as determined by Western blotting) and fibrosis scores.

To elucidate the physiological impact of NF-κBIZ up-regulation during aging, we examined changes induced at the molecular and microscopic levels by aging. It has been well established renal aging is associated with structural changes, such as, renal fibrosis, and that IL-6 and MCP1 are associated with many fibrotic diseases. GO (Gene Ontology) analysis of young and old rat kidneys also revealed that responses of wounding and inflammatory response genes were most changed during aging (Supplementary Table 4). Given renal aging is associated with structural changes in kidneys and that NF-κBIZ-related IL-6 and MCP-1 are fibrotic factors, we investigated the effects of NF-κBIZ up-regulation on age-associated progressive renal fibrosis. Initially, we investigated age-related renal fibrosis in young and old rat kidneys, staining kidney cortices and medullae with Sirius-red and Masson's Trichrome (Figure 5A). Positively stained fibrotic regions in cortices and levels of fibrosis in medullae increased with aging (Figure 5A). Sirius-red and Masson's Trichrome staining also showed fibrotic positive areas were greater in old rat kidneys (Figure 5B). Extracellular matrix proteins, which are known to be abnormally elevated in fibrotic tissues, were also detected in the kidneys of old rats. In fact, the expressions of pro-collagen1, collagen1, and pro-collagen3 were only detected in aged kidneys (Figure 5C), and fibronectin (FN) levels were elevated (Figure 5C), indicating age-associated renal fibrosis had occurred, which concurs with previous reports [16]. In addition, a correlation was found between severity of fibrosis (areas stained by Sirius red) and NF-κBIZ expression levels (R^2^ = 0.8038, Figure 5D).

### Suppression of TGFβ-induced kidney fibroblast activation by knock-down of macrophage NF-κBIZ

**Figure 6:**
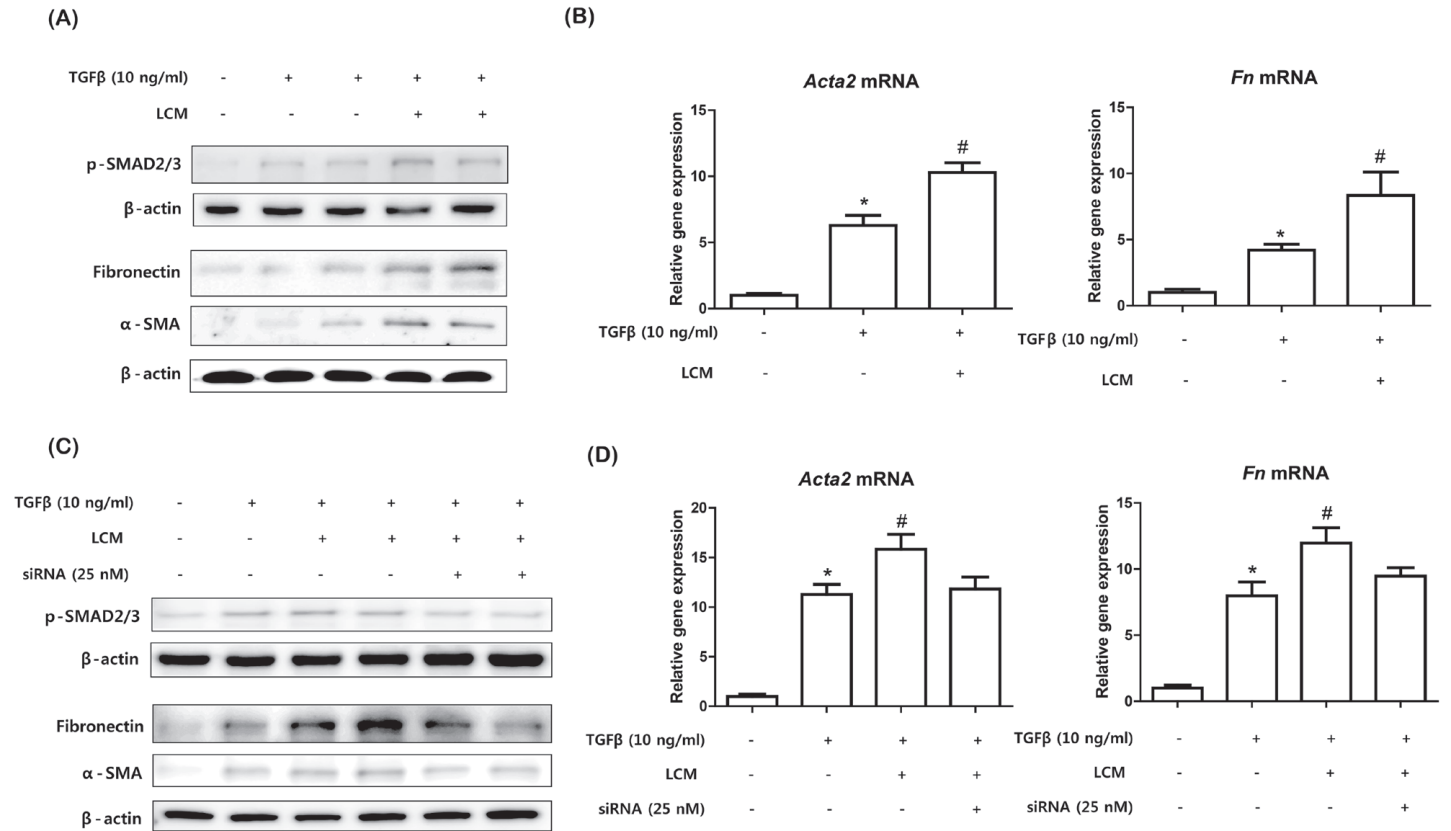
Knock-down of NF-κBIZ in macrophages reduced TGFβ-induced kidney fibroblast activation **A**. Kidney fibroblasts were treated with 1 ng/ml of TGFβ with or without LPS-conditioned macrophage medium. Western blotting was performed to detect SMAD-2/3 phosphorylation and the protein levels of α-SMA and FN in kidney fibroblasts. β-Actin was used as loading control. **B**. qPCR were performed to detect increases in the mRNA levels of *Acta2* and *Fn* in fibroblasts. qPCR results were normalized *versus* *Gapdh*. Data are expressed as means ± SEMs. *P < 0.05 *vs*. non-treated fibroblasts. #*P* < 0.05 *vs*. TGFβ treated fibroblasts. LCM, LPS-conditioned medium. **C**. NF-κBIZ knocked-down kidney fibroblasts were treated with 1 ng/ml of TGFβ with or without LPS-conditioned macrophage medium. Western blotting was used to detect phosphorylated SMAD-2 and to assess the protein levels of α-SMA and FN. β-Actin was used as the loading control. **D**. qPCR was performed to detect the mRNA levels of *Acta2* and *Fn* in fibroblasts. qPCR results were normalized *versus* *Gapdh*. Data are expressed as means ± SEMs. *P < 0.05 *vs*. non-treated fibroblasts. #*P* < 0.05 *vs*. TGFβ treated fibroblasts. LCM, LPS-conditioned medium.

To determine the effects of the up-regulations of NF-κBIZ and related cytokines on fibrosis, we used mouse primary kidney fibroblasts. TGFβ plays pivotal role in many fibrotic diseases by activating fibroblasts to myofibroblasts, which predominate in fibrotic regions. As shown in Figure 6A, TGFβ treatment significantly increased its downstream signaling, as evidenced by the phosphorylation of SMAD, and increased expressions of α-SMA and FN (Figure 6A, 6B). To determine whether peritoneal macrophage derived cytokines promote the activation of fibroblasts induced by TGFβ, we used the medium of peritoneal macrophages stimulated with LPS. Although LPS-conditioned medium did not influence fibroblast activation (data not shown), TGFβ treatment and LPS-conditioned medium significantly increased the downstream signaling of TGFβ (Figure 6A). In addition, LPS conditioned medium significantly increased TGFβ-induced fibroblast activation based on increases in α-SMA and FN levels (Figure 6A, 6B).

Finally, we investigated the effects of NF-κBIZ and related cytokines on fibroblast activation. LPS conditioned medium from NF-κBIZ deficient macrophages had no effect on the TGFβ-induced activation of fibroblasts (Figure 6C, 6D), indicating NF-κBIZ related cytokines (IL-6 and MCP-1) caused the observed increase in fibroblast activation (Figure 6C, 6D). These findings show the role played by NF-κBIZ and related cytokines in TGFβ-induced fibroblast activation.

## DISCUSSION

Previous studies have demonstrated NF-κBIZ plays critical roles in macrophages, NK cells, and T cells [8, 9, 22, 23]. More recent studies have focused of the physiological and pathological roles of NF-κBIZ in relation to immunity and inflammation [25, 26]. However, although the roles played by NF-κBIZ in several immune-related diseases have been extensively researched, the present study is first to describe the role of NF-κBIZ during aging. We first observed that NF-κBIZ was up-regulated during aging by using RNA-sequencing analysis to analyze NGS data. Further biochemical analysis confirmed the importance of NF-κBIZ and its related signaling in aged rat kidneys. Oxidative stresses were found to be associated with increased NF-κBIZ activation in macrophages and in a LPS-treated aged rat kidney model. Based on our interpretations of bioinformatics data and biochemical results, we describe the role played by NF-κBIZ during age-associated renal fibrosis. Furthermore, we believe this investigation provides potential targets for the treatment of age-associated progressive renal fibrosis.

The molecular basis of aging-related inflammation highlights the importance of the molecular events underlying chronic inflammation in aging and age-related diseases [21]. Chronic inflammation has been blamed for renal diseases in the elderly [26]. A study on experimental and human renal disease showed the involvement of inflammation in renal fibrosis induced by diverse diseases process, including aging [18]. In the context of renal inflammation, NF-κB is a key player in the pathogenesis of renal disease whether aging related or not [27]. Previous studies have revealed the importance of NF-κB in the aging process, especially in aged kidneys [20] and brain [28]. Furthermore, because NF-κB is tightly regulated at multiple levels [29], even by other NF-κB family members, these regulators are also interesting targets in terms of the regulation of NF-κB signaling.

NF-κBIZ has been recently identified and implicated in the differential expressions of NF-κB targeted genes in immune cells. Upon activation, NF-κBIZ interacts with the p50 unit of NF-κB, and thus, regulates its transcriptional activity [8]. Importantly, in the present study, the NF-κBIZ gene was the only NF-κB family member found to be up-regulated by RNA-Seq using NGS data. In addition, NF-κBIZ related cytokines (IL-6 and MCP-1) were also found to be up-regulated in aged rat kidney by RNA-Seq analysis which were further verified by biological experiments. Further analysis revealed that NF-κBIZ co-localized with infiltrating macrophages in old rats, indicating that NF-κBIZ elevation during aging is associated with increased macrophage invasion. Interestingly, NF-κBIZ up-regulation was recently reported in a senescent-associated secretory fibroblast phenotype and blocking NF-κBIZ activation prevented senescence [30]. Since senescent-associated cytokines and macrophages play pivotal roles in the regulation of renal inflammation and aging, these findings hint at the physiological meaning of the up-regulations of NF-κBIZ at the transcription and protein levels in aged rat kidneys, and place focus on its pathophysiological role in macrophages.

Renal fibrosis is the final common pathway of all kidney diseases leading to chronic kidney disease [11]. Although the outcomes of renal fibrosis have been well-established, fibrogenesis and the factors that contribute to the progression of fibrosis have yet to be elucidated [13]. Nevertheless, of the many factors responsible for kidney fibrogenesis, aging has been established to be a crucial reason for renal structural changes [17]. Furthermore, because aging is associated with prolonged inflammation, aging promotes chronic inflammation and the pathogeneses of various kidney diseases, including fibrosis [31].

Cytokines are known to play complex roles in fibrosis [32]. In particular, accumulating evidence suggests that IL-6 and MCP-1 play pivotal roles. Although IL-6 is recognized as a pleiotropic cytokine, several studies have revealed its importance in fibrotic diseases. In particular, IL-6 was found to enhance TGF-β induced SMAD activation in fibrosis [33, 34]. Increased IL-6 signaling up-regulated the trafficking of TGF-β receptors to non-lipid raft-associated pools leading to enhanced TGF-β signaling [33]. Furthermore, activation of STAT3-dependent pathways by IL-6 trans-signaling enhanced TGF-β signaling of fibrosis [35]. Most importantly, IL-6 signaling was observed to drive fibrosis signaling in unresolved inflammation by inducing Th1 cell response [36]. STAT3 is also implicated in several fibrotic kidney diseases [37]. Interestingly, STAT3 was implicated as multi-organ target for tissue fibrosis which is not restricted to kidney fibrosis [38, 39]. MCP-1 has been implicated to participate in several types of fibrosis, including renal fibrosis [40, 41]. Although the mechanism has not been precisely identified, the profibrotic actions of MCP-1 are believed to reflect its role in mononuclear cell recruitment and activation. The present study also indicates that these cytokines are associated with renal fibrosis. Cytokines derived from macrophages stimulated with LPS were found to enhance TGF-β induced fibroblast activation *in vitro*. Furthermore, knockdown of NF-κBIZ decreased macrophage productions of IL-6 and MCP-1, and reductions in IL-6 and MCP-1 levels in macrophages achieved by NF-κBIZ knock-down diminished these fibrogenic effects. These findings suggest that NF-κBIZ dependent cytokines play important roles on fibroblasts activation.

In conclusion, the present study demonstrates a new role for NF-κBIZ in age-associated progressive renal fibrosis (summarized in Supplementary Figure 4). NF-κBIZ expression was found to be up-regulated by RNA-Seq analysis, and IL-6 and MCP-1 (both NF-κB target genes regulated by NF-κBIZ) were observed to be significantly up-regulated in aged rat kidneys. Furthermore, the up-regulation of NF-κBIZ in aged rat kidneys was associated with increased macrophage infiltration. Further experiments showed that oxidative stress was associated with increased expression of NF-κBIZ in LPS-stimulated macrophages and kidney tissues. In addition, increased NF-κBIZ expression in aged kidneys was found to be related to the severity of renal fibrosis. Experiments in peritoneal macrophages and primary kidney fibroblasts demonstrated the important role of NF-κBIZ in the acceleration of fibroblast activation induced by TGF-β in fibroblasts. Taken together, this study demonstrates NF-κBIZ is up-regulated in aged rat kidneys and suggests the importance of NF-κBIZ in age-associated progressive renal fibrosis.

## MATERIALS AND METHODS

### Materials

LPS (*Escherichia coli* serotype O111:B5) was purchased from Sigma-Aldrich (Sigma-Aldrich, St. Louis, MO, USA). Antibodies were obtained from Santa Cruz Biotechnology (Santa Cruz, CA, USA), Cell Signaling Technology (New England BioLabs, Hertfordshire, UK), or Abcam (Cambridge, MA, USA). Primers for qPCR were synthesized by Bioneer, Inc. (Daejeon, Korea). Polyvinylidene difluoride (PVDF) membranes were obtained from Millipore (Bedford, MA, USA). Sterile plastic ware for tissue culture was purchased from SPL (Seoul, Korea). All other reagents were purchased from Sigma if not otherwise stated.

### Animals

RNA-Seq data obtained by NGS analysis were obtained from previous results [42]. To assess the biological and biochemical effects of aging on renal inflammation and fibrosis, male Sprague Dawley (SD) rats aged 6 months (young) and 24 (old) months (Samtako, Osong, Korea) were used. Rats were maintained under controlled environmental conditions under a 12-h/12-h light/dark cycle, and allowed *ad libitum* access to water and a standard laboratory diet. Serum was collected for biochemical analysis. Kidneys were collected and either frozen immediately in liquid nitrogen for quantitative polymerase chain reaction (qPCR), Western blot, and biochemical tests, or fixed in neutral-buffered formalin for histochemical examination. The animal protocol used in this study was reviewed and approved of by the Pusan National University-Institutional Animal Care and Use Committee (PNU-IACUC) with respect to study ethicality.

To investigate the effects of aging on LPS-induced renal inflammation. Lipopolysaccharide (LPS, 2 mg/kg body weight) was injected intraperitoneally into young and old male SD rats. Animals were euthanized at 12h after injection. Serum and kidneys were collected for biochemical analysis.

### Cell culture experiments

Primary cultured mouse peritoneal macrophages and primary cultured mouse kidney fibroblasts were used to evaluate the relationship between oxidative stress and NF-κBIZ activation and the role played by NF-κBIZ related cytokines in fibroblast activation. Peritoneal macrophages were obtained from mouse peritoneal, as described previously. Briefly, 5 ml of 3% thioglycollate medium was injected into the peritoneal cavities of C57/BL6 mice, and 4 days later, peritoneal macrophages were collected with a syringe and needle, seeded in cell culture dishes for further experiments. LPS was treated according to experiment's condition. Primary cultured mouse kidney fibroblasts were obtained from Cell Biologics (Chicago, IL). Cells were cultured on gelatin-based coating solution (Sigma-Aldrich, St. Louis, MO), and activated with 2 ng/ml of TGFβ (eBioscience, San Diego, CA). To observe the effects of NF-κBIZ on fibroblast activation, macrophage medium derived supernatants were transferred to fibroblast medium.

### Protein extraction from tissues and cells

All solutions, tubes, and centrifuges were maintained at 0-4°C. PRO-PREP protein extraction solution (Intron Biotech Inc., Korea) was used to extract total protein lysates from tissues or cells according to the manufacturer's instructions. To extract nuclear protein, tissues or cells were washed with ice-cold PBS, suspended in 10 mM Tris (pH 8.0) containing 1.5 mM MgCl_2_, 1 mM dithiothreitol, 0.1% NP-40, and protease inhibitors, homogenized, and incubated on ice for 15 min, and centrifuged at 14,000 *g* for 15 min at 4°C. Supernatants were used as cytosolic fractions and pellets were re-suspended in 10 mM Tris (pH 8.0) containing 50 mM KCl, 100 mM NaCl, and protease inhibitors, incubated on ice for 30 min, and centrifuged at 14,000 *g* for 30 min at 4°C. The resultant supernatants were used as nuclear fractions.

### Western blotting

Western blot assays were performed as described previously with minor modification [19]. Briefly, nuclear or cytosolic proteins (20 ~ 100 μg of protein) were boiled for 5 min in gel-loading buffer (0.125 M Tris-HCl, pH 6.8, 4 % SDS, 10 % 2-mercaptoethanol, and 0.2 % bromophenol blue) at a volume ratio of 1:1. Samples containing the same amounts of proteins were then separated by sodium dodecyl sulfate-polyacrylamide gel electrophoresis in 8 % ~ 15 % acrylamide gels and transferred using a Bio-Rad western system (Bio-Rad, Hercules, CA, USA) to PVDF membranes, which were immediately placed in blocking buffer (5% non-fat milk) containing 10 mM Tris (pH 7.5), 100 mM NaCl, and 0.1% Tween 20. Membranes were then washed in TBS-Tween buffer for 30 min, incubated with specific primary antibodies (dilution 1:500 to 1:2000, Table 1) at 4°C overnight, washed for 3×10-min in TBS-Tween buffer, and incubated with horseradish peroxidase-conjugated anti-mouse antibody (Santa Cruz, 1:10,000), anti-rabbit antibody (Santa Cruz, 1:10,000), or anti-goat antibody (Santa Cruz, 1:10,000) at 25°C for 1 h. Resulting immunoblots were visualized using Western Bright Peroxide solution (Advansta, CA, USA) and Davinch-chemi CAS-400 (Davinch-K, Seoul, Korea), according to the manufacturers’ instructions.

### Isolation of total RNA and qPCR

Total RNA was isolated as previously described. Briefly, tissue samples were homogenized in the presence of RNAzol^TM^ (2 ml per 100 mg tissue) with a few strokes in a tissue homogenizer. Aliquots of 0.2 ml chloroform per 2 ml homogenate were added and samples were shaken vigorously for 15 min. Aqueous phases were transferred to fresh tubes, to which an equal volume of isopropanol was added. Samples were then left at 4°C for 15 min and centrifuged at 12,000 *g* at 4°C for 15 min. Supernatants were removed and RNA pellets were washed once with 75% ethanol by vortexing and then centrifuged at 7,500 *g* at 4°C for 8 min. Pellets were dried for 10-15 min and dissolved in DEPC-treated water. RNase-free DNase-treated total RNA (2.0 μg) was reverse-transcribed using a cDNA synthesis kit from Gendepot (Barker, TX, USA). DEPC-treated water and 250 ng of random primer were added, incubated at 75°C for 5 min, and then on ice for 5 min. Aliquots of 2 μl of 0.1 M DTT, 4 μl of 5X buffer, 4 μl of 2.5 mM dNTP, 100 U of reverse transcriptase, and 16.5 U of RNase inhibitor were added and the mixture was incubated at 37°C for 2 h. The reaction was stopped by boiling at 100°C for 2 min, and the cDNA so obtained was stored at -20°C until required. qPCR was performed using Sybr-Green and the CFX Connect System (Bio-Rad Laboratories Inc., Hercules, CA, USA). Primer sequences are provided in Supplementary Table 5.

### Histopathological analysis

Kidneys were fixed in 10% neutral formalin and paraffin-embedded sections were stained with hematoxylin and eosin (H&E). Sirius-red staining and Masson's Trichrome staining were performed to determine the degree of aging-associated fibrosis, as described previously [43]. Immunohistochemical staining for kidney macrophages was performed using ED-1 antibody.

### Measurement of oxidative stress

RS generation was measured using 2′,7′-dichlorodihydro-fluorescein diacetate (DCFDA; a fluorescent probe). For tissue homogenates, 25 μM DCFDA was added in 50 mM of phosphate buffer. Changes in fluorescence intensity were measured every 5 min for 30 min on a microplate reader (GENios; Tecan Instruments, Salzburg, Austria) using excitation and emission wavelengths of 485 and 530 nm, respectively. For cell culture experiments, after cells had been treated, they were incubated with 10 μM carboxy-H_2_DCFDA for 10 min at 37°C and washed twice with PBS. Dye oxidation rates were determined by monitoring changes in fluorescence intensity every 5 min for 30 min using a fluorescence plate reader using excitation and emission wavelengths of 485 and 530 nm, respectively.

### Immunofluorescence

Paraffin-embedded young and old kidney sections were deparaffinized, rehydrated and retrieved. After blocking with 5% BSA in PBS for 30 min, sections were incubated in a mixture of two primary antibodies overnight at 4°C. After washing with PBS, sections were incubated with the mixture of two secondary antibodies raised in different species [Alexa Fluor-488 Goat Anti-rabbit IgG (H+L) Antibody (Invitrogen) and Rhodamine RedTM-X Goat Anti-Mouse IgG (H+L) antibody (Jackson ImmunoResearch, West Grove, PA, USA)] in 1% BSA for 2 h at room temperature in the dark. Sections were then counterstained with 1 μg/ml Hoechst 5 min, mounted, and stored in dark at 4°C. Confocal images were obtained using a FV10i FLUOVIEW Confocal Microscope (Olympus, Tokyo).

### siRNA transfection

The siRNAs of NF-κBIZ and negative controls were purchased from Integrated DNA Technologies (IDT, Coralville, IA, USA). Dicer-substrate siRNA methods were used to increase the potency of target gene knock-down. The selected NF-κBIZ siRNA-targeting sequence (duplex1 in experiment) was AUAUUUGUAGUUCUUACUCACGUUGGG. TYE-563 fluorescently-labeled transfection control duplex was used for detecting the efficiency of transfection in peritoneal macrophages. Lipofectamine 3000 (Invitrogen) was used to transfect cells with siRNA. For siRNA transfection, cells were seeded in six-well plates, grown for 24 h to 60-70% confluence, and then transfected with 25 nM NF-κBIZ siRNA or negative control siRNA using Lipofectamine 3000 (Invitrogen).

### Statistical analysis

Analysis of variance (ANOVA) was used to analyze intergroup differences. Differences between group means were assessed using Fisher's protected least-significant difference *post hoc* test. The Student's *t*-test was used to analyze differences between two groups. *P* values of < 0.05 were considered statistically significant. Analyses were performed using GrapdPad Prism 5 (GraphPad software, La Jolla, CA).

## SUPPLEMENTARY MATERIALS FIGURES AND TABLES












