# Supplementary material for: Involvement of NF-κBIZ and related cytokines in age-associated renal fibrosis
Source: Oncotarget. 2017 Jan 12;8(5):7315–27. doi: 10.18632/oncotarget.14614 (PMC5352323; doi:10.18632/oncotarget.14614)
Supplement: Supplementary file 1 [file oncotarget-08-7315-s001.pdf]

Involvement of NF-κBIZ and related cytokines in age-associated renal fibrosis

Supplementary Table 1. RNA-sequencing analysis data of NF-κB family members.
Supplementary Table 2. RNA-sequencing analysis data of NF-κB target genes.
Supplementary Table 3. NF-κB target genes found to be significantly changed by RNA-sequencing analysis.
Supplementary Table 4. The importance of fibrosis related genes by gene ontology analysis in young and old rat kidneys.
Supplementary Table 5. Primers used for qPCR.

For Supplementary Tables see on Supplementary Information.

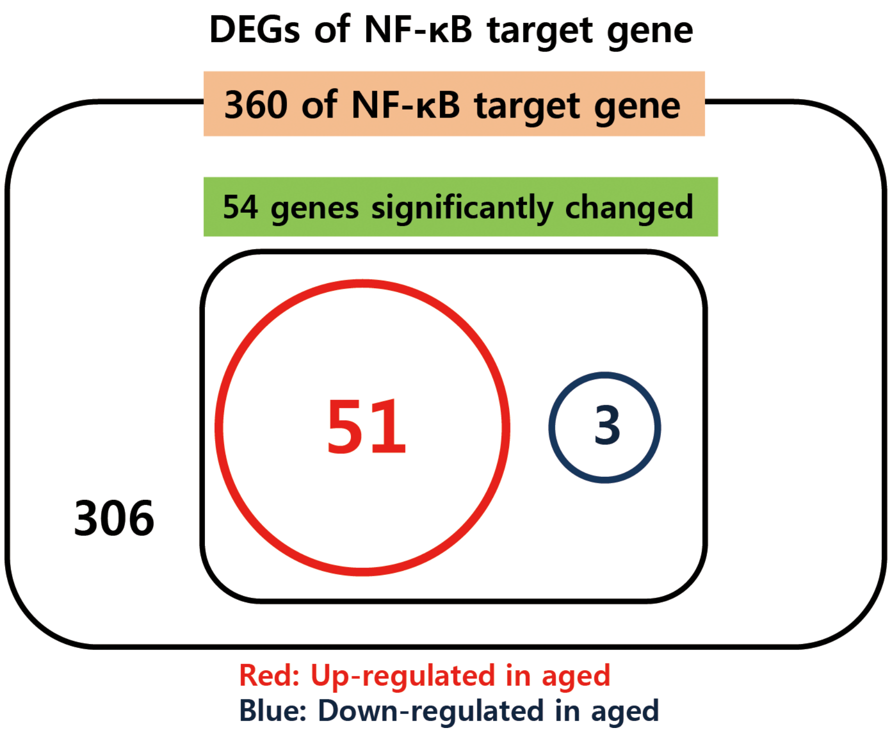

Supplementary Figure 1: Schematic of RNA-sequencing analysis using NGS data.

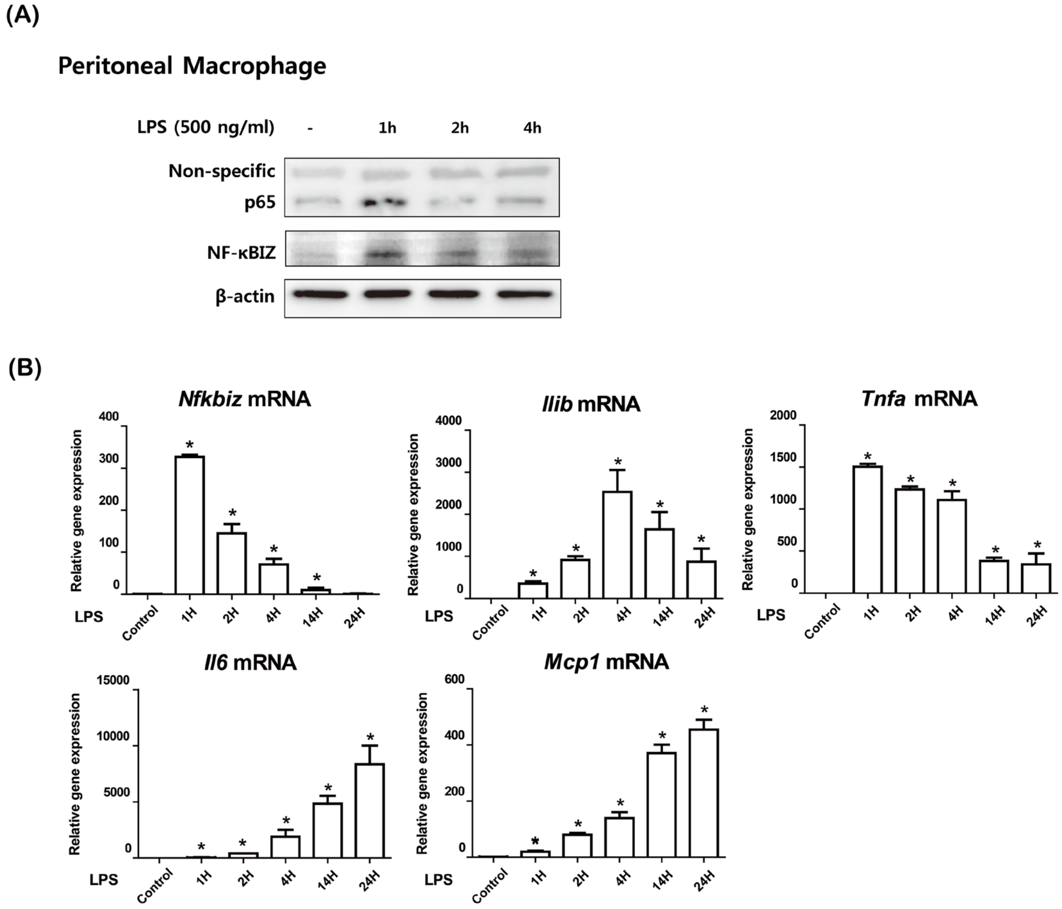

Supplementary Figure 2. Enhanced LPS mediated NF-κB signaling in peritoneal macrophages

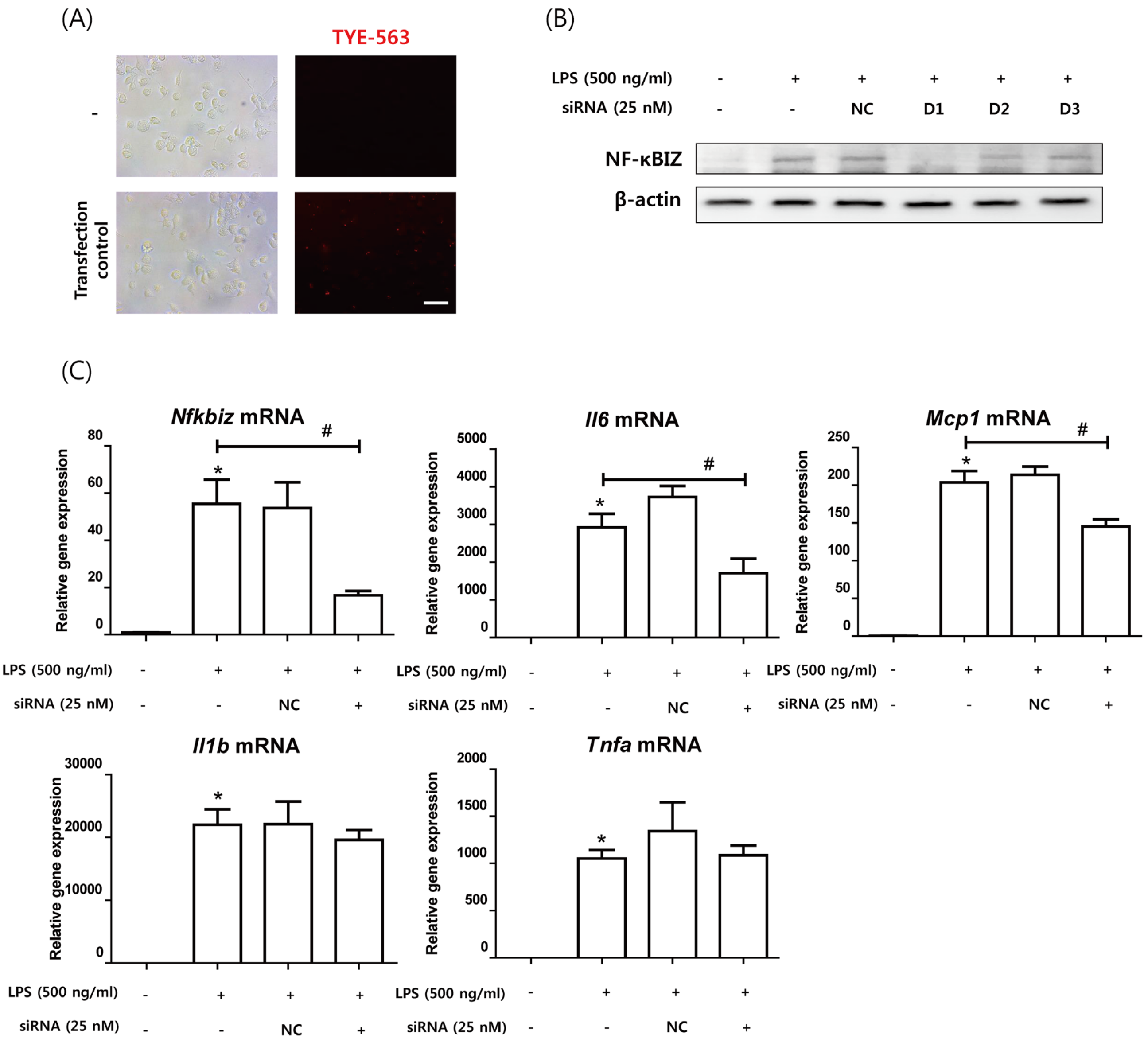

Supplementary Figure 3. Modulations of IL-6/MCP-1 by NF-κBIZ in LPS-treated peritoneal macrophages.

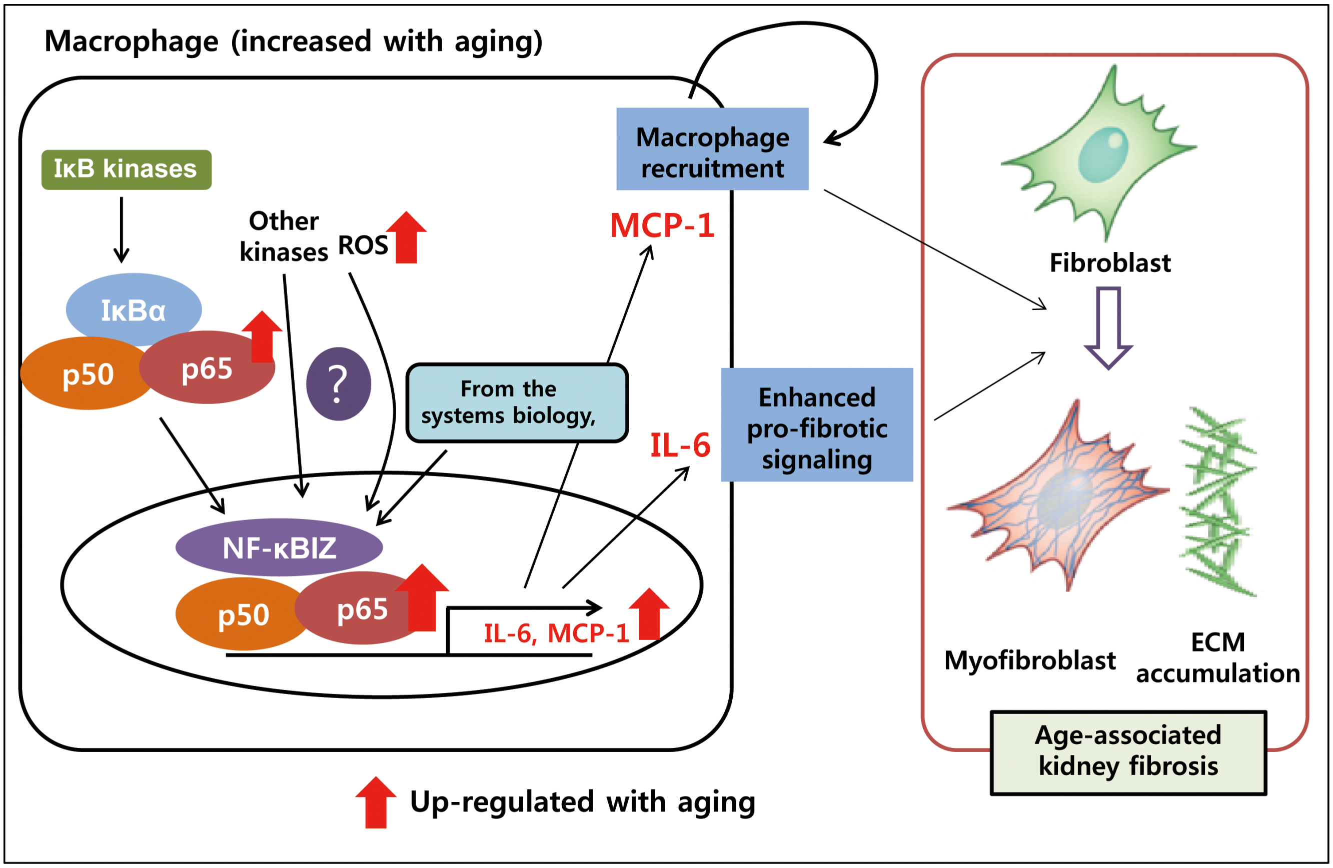

Supplementary Figure 4. Proposed effects of NF-κBIZ on age-associated progressive renal fibrosis.
